# SEMU-Net: A Structure-Enhanced Multi-Branch U-Shaped Network for High-Resolution Remote Sensing Land-Cover Segmentation

**DOI:** 10.3390/s26165290

**Published:** 2026-08-20

**Authors:** Bingyan Lu, Mei Li, Xiaorong Xue, Wen Zhang, Xin Zhao, Jingtong Yang, Yishuo Tian, Wancheng Wang

**Affiliations:** 1 School of Electronics and Information Engineering, Liaoning University of Technology, Jinzhou 121001, China; lubingyan2332@163.com (B.L.); z13477988185@163.com (W.Z.); 15242191552@163.com (X.Z.); 13188937083@163.com (J.Y.); tian761002@163.com (Y.T.); yike8191@163.com (W.W.); 2 School of Urban and Environmental Sciences, Peking University, Beijing 100871, China; mli@pku.edu.cn

**Keywords:** high-resolution remote sensing imagery, semantic segmentation, multiscale feature fusion, channel modulation mechanism, U-shaped network

## Abstract

High-resolution remote sensing semantic segmentation remains challenging because repeated downsampling progressively weakens the fine-grained spatial information of small objects, while direct fusion of heterogeneous multi-scale features may introduce semantic discrepancies and redundant background responses. To address these issues, this study proposes SEMU-Net, a structure-enhanced multi-branch U-shaped network. First, an independent multi-scale complementary branch is constructed outside the main encoder pathway to provide auxiliary hierarchical representations and compensate for information degradation during progressive semantic abstraction. Second, a scale-consistent feature embedding module is introduced to project and normalize side-branch features before residual injection, thereby improving the compatibility of cross-path feature representations. Third, a discriminative channel modulation module is incorporated into the decoder to adaptively strengthen task-relevant channel responses and suppress redundant background activations. Experiments were conducted on the ISPRS Vaihingen dataset and a self-annotated high-resolution remote sensing dataset. On the Vaihingen dataset, SEMU-Net achieved a mIoU of 72.21% and an Average F1 score of 83.64%, outperforming the strongest competing method by 0.59 and 0.44 percentage points, respectively. The IoU of the Car class increased by 3.50 percentage points. On the self-annotated dataset, the IoU of the narrow Road class improved by 4.12 percentage points. These results demonstrate that SEMU-Net improves overall segmentation accuracy and enhances the recognition of small objects, with the observed improvements being consistent with the design objectives of multi-scale information compensation, cross-path feature adaptation, and channel recalibration.

## 1. Introduction

**Figure 1:**
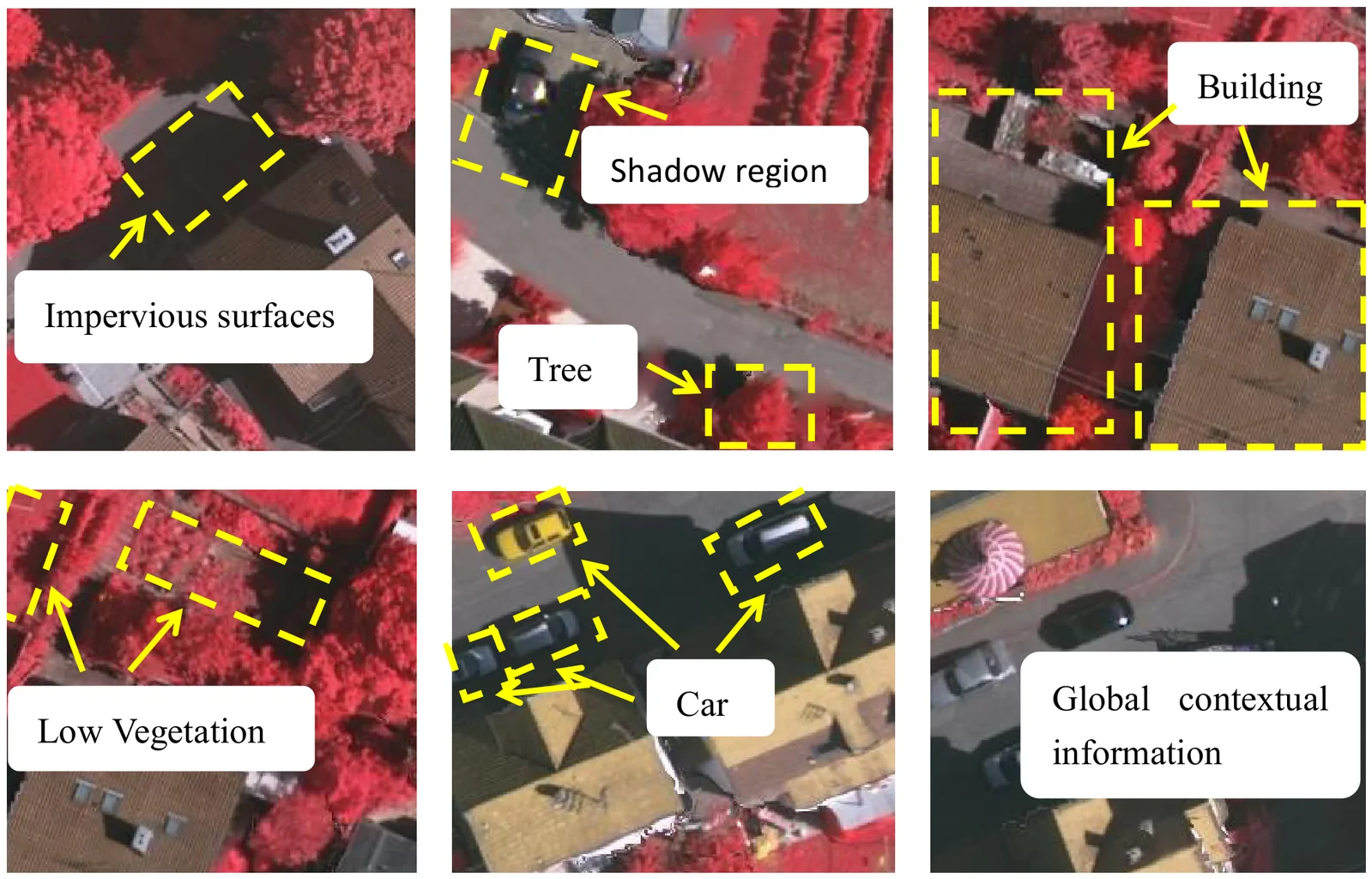
Typical challenges in the ISPRS Vaihingen dataset. Small objects (e.g., cars) are easily suppressed during hierarchical abstraction. Vegetation is often affected by occlusion and shadows. Impervious surfaces and buildings exhibit high spectral similarity, leading to local semantic ambiguity and spatial inconsistency.

High-resolution remote sensing image semantic segmentation is a key technology for land-cover mapping [1], land-cover change detection and sub-pixel mapping [2], fine-grained urban management [3], ecological environment monitoring [4], and resource scheduling and decision-making [5]. With the continuous improvement of spatial resolution in Earth observation systems, remote sensing imagery exhibits increasingly complex texture patterns and spatial structures [6]. Compared with natural scene images, high-resolution remote sensing images are characterized by more pronounced scale variations, more intricate spatial relationships among objects, and higher inter-class spectral similarity, often resulting in densely distributed small objects, fragmented regions, and local semantic ambiguity. Figure 1 shows an IRRG color-infrared image from the Vaihingen dataset. In this image, small-scale objects such as vehicles are often overwhelmed by complex backgrounds, while buildings and impervious surfaces exhibit highly similar spectral and textural responses. In addition, shadows and projection regions further exacerbate semantic ambiguity. Under such complex conditions, semantic segmentation models often struggle to preserve fine-grained spatial information and maintain consistent semantic predictions across different scales.

Specifically, high-resolution remote sensing semantic segmentation still faces two major challenges. On the one hand, the fine-grained spatial information of small and elongated objects is progressively weakened during repeated downsampling, which may lead to missed or incomplete predictions for objects such as vehicles and narrow roads [7]. Qureshi et al. showed that aerial vehicle analysis remains sensitive to object-scale variation and complex background interference [8]. To address the weak representation of small targets, Li et al. further proposed a ConvNeXt-based U-shaped architecture with slicing-aided hyper-segmentation to enhance small-object representation [9]. On the other hand, complex spatial adjacency, together with high spectral and textural similarity among land-cover classes, may produce ambiguous feature responses in local transition regions, making it difficult to maintain region-level semantic consistency [10].

These challenges are closely related to the hierarchical feature extraction and cross-level fusion mechanisms employed in prevailing segmentation networks. Convolutional neural networks based on encoder–decoder architectures have become the mainstream framework for remote sensing semantic segmentation. Such methods progressively enlarge the receptive field and extract high-level semantic features during the encoding stage, and they then recover spatial details and reconstruct semantic structures in the decoding stage through skip connections that fuse shallow spatial features. From the perspective of multi-scale modeling, the hierarchical abstraction in the encoder enables the network to gradually perceive semantic information at different scales, while skip connections supplement fine-grained structural details by introducing high-resolution features. However, this form of multi-scale modeling still largely relies on a single-path hierarchical propagation mechanism. During continuous downsampling, shallow textures and fine-grained spatial cues are progressively weakened, limiting the representation capability for small-scale objects. Meanwhile, skip connections typically adopt direct concatenation or element-wise addition, with limited adaptation to representational discrepancies across different scales. In complex remote sensing scenes, such fusion strategies may introduce cross-scale representation mismatch, feature redundancy, and semantic interference, resulting in unstable predictions and fragmented regions, particularly for small objects and semantically heterogeneous transition regions.

To overcome the above limitations of single-path hierarchical propagation and direct skip fusion, recent studies have introduced multi-branch architectures and attention-based fusion mechanisms. These methods typically employ parallel pathways to extract complementary representations from different receptive fields or semantic levels and use attention weights to emphasize task-relevant responses [11]. Although these designs diversify feature sources and improve feature selection, their auxiliary features are often derived from intermediate backbone representations or fused through direct concatenation, addition, or weighted aggregation. Consequently, they mainly enhance or reorganize features that have already undergone hierarchical abstraction and may not provide an independent source of fine-grained information compensation outside the main downsampling pathway.

Furthermore, features generated by different pathways or representation processes may exhibit discrepancies in semantic granularity and statistical distribution. Related studies on fine-grained remote sensing segmentation have also highlighted the difficulty of transferring and effectively utilizing detailed semantic information across heterogeneous representations [12]. When heterogeneous features are fused without explicit adaptation, direct concatenation or weighted aggregation may introduce redundant responses and semantic interference. Therefore, increasing the number of branches alone does not necessarily guarantee effective cross-path collaboration.

Methods that integrate multi-source or multimodal representations further highlight the importance of coordinating heterogeneous feature spaces in semantic segmentation [13]. These studies indicate that effective fusion depends not only on increasing feature diversity but also on appropriately coordinating representations generated by different sources or pathways. However, simple feature aggregation does not necessarily resolve discrepancies in channel dimensionality, semantic granularity, and activation statistics. Together with the limitations of existing multi-branch methods discussed above, this observation suggests that an effective framework should provide an additional source of complementary information, adapt heterogeneous representations before fusion, and refine the fused responses afterward.

In summary, although existing methods have achieved considerable progress in multi-scale representation and feature enhancement, two limitations remain. First, fine-grained information for small objects is progressively weakened during repeated downsampling, whereas many auxiliary representations remain coupled with the backbone abstraction process. Second, heterogeneous cross-path features are often fused without sufficient adaptation of their channel representations, semantic granularity, and activation statistics, which may introduce redundancy and semantic interference. These limitations motivate the independent compensation and scale-consistent feature adaptation adopted in SEMU-Net.

The main contributions of this article are summarized as follows:

(1) A structure-enhanced multi-branch U-shaped framework is proposed. An independent multi-scale compensation pathway is constructed alongside the backbone encoder–decoder architecture. Unlike auxiliary features derived from intermediate backbone states, this pathway directly builds hierarchical representations from the input space, allowing fine-grained spatial information to continuously participate in subsequent semantic reconstruction and alleviating information degradation for small objects during repeated downsampling.
(2) A scale-consistent feature mapping mechanism is designed. To address potential inconsistencies in channel representation, semantic granularity, and activation statistics between cross-path features, the proposed mechanism performs channel projection and normalization before residual injection. This design improves the representation compatibility between side-branch and backbone features and enables cross-path feature fusion without additional channel expansion.
(3) A post-fusion discriminative channel modulation module is incorporated. Through global semantic compression and adaptive channel recalibration, DCM strengthens task-relevant channel responses and suppresses redundant background activations, thereby improving the discriminative capability of the fused representation.

## 2. Related Works

### 2.1. Multiscale Semantic Modeling Based on Convolutional Networks

Convolutional neural networks (CNNs) have long been the dominant framework for semantic segmentation of remote sensing images. The fully convolutional network (FCN) proposed by Long et al. [14] was the first to achieve end-to-end pixel-wise prediction, laying the foundation for dense segmentation tasks. Subsequently, Audebert et al. [15] introduced FCN into high-resolution remote sensing image segmentation, which significantly promoted the systematic application of deep learning methods in the remote sensing domain.

In high-resolution remote sensing scenarios, different land-cover objects often exhibit significant scale variations [16]. Therefore, how to effectively model multi-scale semantic features during feature extraction has become a key research challenge. U-Net, proposed by Ronneberger et al. [17], adopts an encoder–decoder architecture with skip connections to fuse low-level and high-level features. This design combines high-level semantic information with high-resolution spatial details, making it a fundamental baseline for remote sensing segmentation. Further improvements upon U-Net have continuously advanced multi-scale feature fusion. For example, UNet++ [18] reduces the semantic gap between features at different levels through dense skip connections, thereby improving cross-scale feature interaction. ResUNet++ [19] enhances representation capability and training stability by incorporating residual structures.

For multi-scale context modeling, researchers have explored enlarging the receptive field to enhance cross-scale semantic representation. DeepLabv3+ [20] employs atrous spatial pyramid pooling (ASPP) [21] to capture contextual information at multiple receptive-field scales and combines shallow features in the decoder to recover fine-grained spatial information. PSPNet [22] aggregates global contextual information at multiple scales through pyramid pooling, while HRNet [23] maintains high-resolution representations throughout the network and continuously exchanges information across scales, thereby alleviating the loss of spatial information caused by repeated downsampling. More recently, SegNeXt [24] enlarged the receptive field using large-kernel convolutions and re-parameterization strategies to strengthen contextual modeling. In addition, feature pyramid structures inspired by FPN [25] and UPerNet [26] propagate semantic information in a top-down manner, enabling progressive multi-scale feature fusion.

Despite these advances, most existing approaches still rely primarily on hierarchical feature propagation within the backbone for multi-scale modeling. During repeated downsampling, shallow texture cues and fine-grained spatial information may gradually degrade, limiting the representation of small-scale and spatially fragmented objects [27,28]. Meanwhile, although deep features encode richer semantic information, their reduced spatial resolution makes it difficult to recover detailed object layouts and local spatial relationships during decoding. Therefore, simply enlarging the receptive field or stacking multi-scale modules may not sufficiently address the degradation of fine-grained information and the incompatibility between features from different paths and scales. These limitations motivate the development of explicit complementary pathways and cross-path feature adaptation mechanisms.

### 2.2. Multiscale Feature Fusion and Compensation Mechanisms

To mitigate the loss of fine-grained information caused by single-path feature abstraction, researchers have proposed multi-path architectures and feature compensation mechanisms to enhance multi-scale feature representation. ABCNet [29] constructs a bilateral path to model local details and global context separately, and it then integrates them through a feature aggregation module. MCCANet [30] adopts a context-aware dynamic feature fusion strategy to enhance intra-class consistency and improve local spatial representation. ERN [31] introduces edge enhancement modules and auxiliary structural loss functions to reduce semantic ambiguity, thereby improving segmentation accuracy in spatially heterogeneous regions.

In remote sensing segmentation, small objects and spatially fragmented land-cover objects heavily depend on high-resolution shallow features. Accordingly, various studies have explored multi-scale compensation strategies to enhance fine-grained structural modeling. For instance, Ma et al. [32] proposed a segmentation framework tailored for small objects, which employs a feature enhancement pyramid (FEP) along with a joint focal loss to address class imbalance and weak representation issues. Relation Matters [33] introduces a relational context-aware network that models long-range pixel dependencies to strengthen feature representation and improve segmentation performance in spatially heterogeneous regions. In addition, remote sensing adaptations based on the Segment Anything Model (SAM) [34] leverage object-consistency and structure-preserving losses to enhance sensitivity to local structural variations and regional consistency.

Furthermore, some works focus on cross-scale feature alignment and recalibration. SKNet [35] adaptively selects convolutional kernels with different receptive field sizes to dynamically modulate feature responses, thereby enhancing multi-scale feature representation capability; other approaches employ 1×1 convolutional projections, normalization, or residual mappings to achieve cross-scale feature matching. Building upon these ideas, recent studies have further explored feature enhancement and fusion optimization. $A^{2}$-FPN [36] introduces adaptive feature modulation within the feature pyramid to strengthen inter-level information interaction, thereby improving cross-scale fusion quality. ESMSNet [37] enhances fine-grained structural modeling through multi-branch feature compensation, while AFENet [38] improves the collaborative representation of high- and low-level features via adaptive feature enhancement. These methods, by incorporating feature recalibration, structural compensation, and cross-level interaction, improve both multi-scale semantic representation and detail preservation to a certain extent.

Beyond land-cover semantic segmentation, related remote sensing tasks have also investigated complementary representation learning and cross-scale feature interaction, providing relevant insights into multi-path feature coordination. In infrared small-target detection, Wang et al. proposed DABF-Net, which combines dual-branch attention guidance with bidirectional feature enhancement to strengthen weak target representations [39]. For cropland parcel extraction, Wu et al. developed CT-HiffNet to integrate complementary contour and texture information through hierarchical feature fusion [40]. Similarly, Fu et al. introduced DFDR-NLNet for photovoltaic-panel segmentation, in which differentiated frequency representations are constructed and exchanged across scales to enhance feature interaction [41]. Although these methods address different application targets, they consistently indicate that complementary feature sources must be effectively coordinated across branches, levels, or representation domains.

Another group of studies focuses on informative-feature selection, heterogeneous representation reorganization, and redundant-response suppression. Wei et al. proposed FSINet for multi-scale SAR object detection, in which feature separation and adaptive feature integration are employed to reduce noise interference and improve multi-scale object representation [42]. Shou et al. introduced a graph information bottleneck into remote sensing semantic segmentation to compress task-independent redundant information in learned representations [43]. From the perspective of heterogeneous spatial representation reorganization, Li et al. developed UE-Extractor for point-cloud ground extraction and employed adaptive grid projection to transform irregular point-cloud representations into a regularized form more suitable for subsequent feature extraction [44]. Despite differences in sensing modality and task formulation, these studies collectively demonstrate the importance of informative-feature selection, representation adaptation, and redundant-response suppression in complex remote sensing feature modeling.

Taken together, existing studies have made substantial progress in multi-branch compensation, cross-scale interaction, adaptive recalibration, and redundant-information suppression. However, these components are generally addressed separately by different methods and have not been fully coordinated within a unified framework. In many multi-scale fusion networks, features from different pathways are still combined through direct concatenation, element-wise addition, or weighted aggregation, while discrepancies in channel representation, activation statistics, and semantic granularity are not always jointly considered. This may introduce incompatible feature responses or unnecessary information redundancy. Therefore, coordinating complementary information construction, pre-fusion representation adaptation, and controlled information injection during feature interaction remains an important challenge in multi-path feature fusion.

### 2.3. Attention Mechanisms, Transformer-Based Modeling, and Structure-Guided Segmentation

Attention mechanisms dynamically recalibrate feature responses, enabling models to focus on critical semantic regions and thereby enhancing feature representation. SENet [45] introduces channel-wise recalibration to strengthen feature representation, while CBAM [46] combines spatial and channel attention to achieve multi-dimensional feature refinement. DANet [47] incorporates both position and channel attention to model long-range dependencies. CCNet [48] further enhances feature interaction by efficiently capturing global contextual dependencies through criss-cross attention. In remote sensing scenarios, MANet [49], SPANet [50], and TANet [51] improve the segmentation of small objects and spatially complex land-cover regions through multi-scale attention or sequential pooling strategies. LANet [52] jointly encodes local and global context through patch attention modules (PAM) and attention embedding modules (AEM).

With the advancement of research, hybrid architectures combining convolutional networks and Transformers have emerged as an effective paradigm for cross-scale modeling. These methods preserve the local modeling capability of CNNs while introducing self-attention mechanisms to enhance global dependency modeling. For example, TransUNet [53] incorporates Transformers into the encoder to strengthen global semantic representation. Swin-UNet [54] leverages hierarchical window-based attention to enable efficient cross-scale interaction. UNetFormer [55] integrates Transformer structures into a U-shaped architecture to achieve multi-scale semantic modeling. CMT [56] combines convolution and self-attention to improve cross-scale feature representation. ST-UNet [57] fuses spatial information with Transformer structures to enhance multi-scale representation, while SCG-TransNet [58] introduces spatial-context guidance to improve cross-scale semantic consistency.

More recently, Vision Transformers (ViT) [59] have provided a new paradigm for modeling long-range dependencies across spatial regions. SETR [60] and Segmenter [61] adopt pure Transformer architectures to directly model patch-level semantic relationships. Swin Transformer [62] reduces computational complexity through shifted-window attention while enabling cross-window information exchange. SegFormer [63] employs a hierarchical design with efficient attention mechanisms for multi-scale semantic modeling. For remote sensing segmentation, DC-Swin [64] utilizes Swin Transformer to extract multi-scale global features and integrates them through dense connections, while SegViT [65] generates segmentation results through an attention-to-mask mechanism.

Although Transformer-based methods are effective in modeling global semantic relationships, their high computational complexity still introduces substantial overhead in high-resolution remote sensing image processing [66]. Meanwhile, fine-grained structural information may gradually degrade during hierarchical propagation, making the recognition of small objects and spatially fragmented land-cover regions strongly dependent on effective local feature representation [67]. In contrast, CNN-based architectures are naturally effective in modeling local textures, fine-grained details, and high-resolution spatial information. Therefore, enhancing multi-scale representation and detail compensation through structural design while retaining efficient local modeling remains an important direction for improving the recognition of small objects and spatially complex land-cover categories.

Beyond attention- and Transformer-based global relation modeling, recent structure-guided approaches have incorporated explicit auxiliary supervision into remote sensing semantic segmentation. BGSC-Net [68] combines a cross-level semantic compensation module with a decoder-driven auxiliary boundary supervision module. The latter aggregates multi-level decoder features within an auxiliary edge branch and jointly optimizes boundary prediction and the main semantic segmentation task. EdgeGuided-Net [69] adopts a PVTv2-based semantic–edge dual-branch architecture, in which a boundary-guided module adaptively integrates semantic and edge information, while a learnable Laplacian operator and a depthwise residual module extract high-frequency cues. These studies demonstrate that auxiliary structural supervision and high-frequency information can complement semantic representations and improve the spatial characterization of land-cover objects. Xie et al. proposed a CNN–Transformer hybrid network for cropland field-parcel mapping [70]. The method combines local–global feature modeling with a boundary-guided enhancement mechanism to improve the representation of parcel-level spatial patterns.

Complementary to explicit structure-guided modeling, another line of research combines multi-branch Transformer architectures with multi-scale feature fusion. MBT-UNet [71] combines a multi-branch Pyramid Vision Transformer encoder with a feature fusion module and a multi-scale upsampling module. This architecture strengthens multi-scale contextual extraction, feature integration, and decoder-side reconstruction.

Taken together, attention mechanisms, Transformer-based models, and structure-guided methods enhance feature representation through adaptive recalibration, contextual modeling, and auxiliary structural information. However, they do not explicitly address the degradation of input-derived fine-grained information during downsampling or the adaptation of heterogeneous cross-path features before fusion. These limitations motivate further exploration of coordinated information compensation and feature adaptation.

### 2.4. Comprehensive Analysis and Research Motivation

Overall, existing remote sensing semantic segmentation methods have advanced along several directions, including convolutional multi-scale semantic modeling, multi-path feature fusion and compensation, attention-based feature recalibration, Transformer-based contextual modeling, and structure-guided segmentation. These approaches improve feature representation from different perspectives, such as preserving spatial information, expanding contextual dependencies, enhancing complementary features, and introducing auxiliary structural cues. However, they generally emphasize individual stages of the segmentation process, while the coordination among complementary information construction, cross-path representation adaptation, and post-fusion feature refinement remains insufficiently explored.

In particular, many auxiliary representations are still derived from intermediate backbone features and therefore remain coupled with the hierarchical abstraction process. Moreover, heterogeneous features from different pathways are often combined through direct concatenation, element-wise addition, or weighted aggregation without sufficient adaptation of their channel representations, semantic granularity, and activation statistics. Such fusion may limit the effective utilization of complementary information and introduce redundant or incompatible responses.

Motivated by these observations, SEMU-Net organizes its information flow into three coordinated stages: input-derived fine-grained information compensation, pre-fusion cross-path feature adaptation, and post-fusion channel modulation. MSCB constructs an auxiliary multi-scale feature hierarchy directly from the input space to provide a complementary information source outside the main downsampling pathway. SFM applies channel projection and normalization before residual injection to improve the compatibility between side-branch and backbone representations. DCM subsequently recalibrates the fused features to strengthen task-relevant channel responses and suppress redundant background activations. Through this functional organization, complementary information construction, representation adaptation, and post-fusion recalibration are coordinated within a unified framework.

## 3. Method

### 3.1. Overview

**Figure 2:**
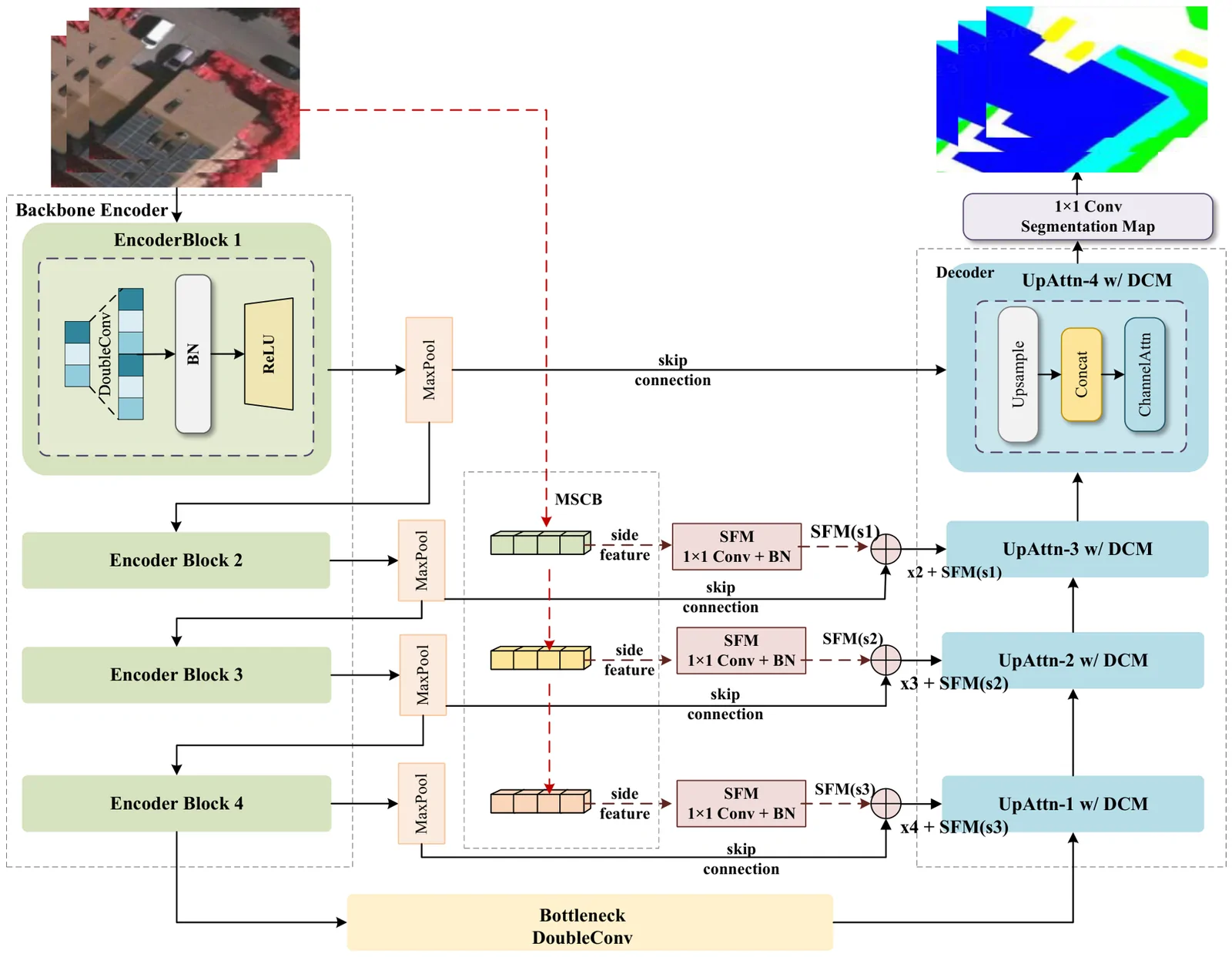
Overall architecture of SEMU-Net. MSCB constructs an input-derived multi-scale complementary pathway, SFM adapts and residually injects side-branch features into the corresponding backbone skip features, and DCM recalibrates the fused decoding representations.

As illustrated in Figure 2, SEMU-Net is constructed upon a U-shaped encoder–decoder architecture to address three limitations in high-resolution remote sensing segmentation: the progressive degradation of fine-grained information during downsampling, the representation discrepancy between heterogeneous feature pathways, and redundant channel responses after feature fusion. The proposed framework therefore organizes its information flow into three coordinated stages: independent multi-scale compensation, cross-path feature adaptation, and post-fusion channel modulation.

First, the Multi-Scale Complementary Branch (MSCB) constructs an auxiliary feature hierarchy directly from the input image at the 1/2, 1/4, and 1/8 resolution levels. Unlike auxiliary representations derived from intermediate backbone states, these features provide an independent source of fine-grained information that complements the progressively abstracted backbone representations.

Second, the Scale-Consistent Feature Embedding Module (SFM) adapts each side-branch feature before fusion. It maps the feature to the channel space of the corresponding backbone skip feature, adjusts its statistical representation, and introduces it through residual addition. This design improves cross-path representation compatibility while avoiding the channel expansion caused by direct concatenation.

Third, the Discriminative Channel Modulation module (DCM) is applied to the decoded feature after skip-feature fusion. It generates channel-wise modulation weights from the current feature representation to strengthen task-relevant responses and suppress redundant activations. Because DCM operates on a single decoding feature tensor, it can process either the backbone-only representation or the representation containing information injected by MSCB and SFM.

The final decoding representation is mapped to the semantic category space through a prediction layer. The distinction of SEMU-Net lies in the coordinated organization of input-derived information compensation, pre-fusion representation adaptation, and post-fusion channel modulation, rather than in modifications to the standard convolutional operations of the U-shaped backbone.

### 3.2. MSCB Module

**Figure 3:**
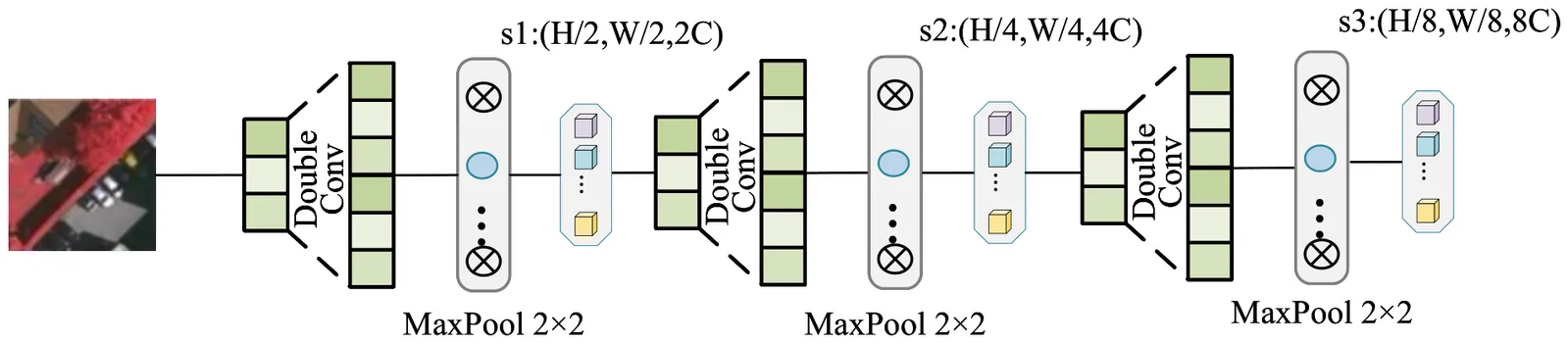
Structure of the Multi-Scale Complementary Branch (MSCB). The branch constructs input-derived auxiliary features at the 1/2, 1/4, and 1/8 resolution levels to complement the corresponding backbone representations.

As illustrated in Figure 3, this study introduces a Multi-Scale Complementary Branch (MSCB) outside the backbone U-shaped topology. Rather than reusing intermediate representations from the backbone encoder, MSCB directly constructs an auxiliary hierarchical representation from the original input and provides complementary features at multiple resolutions for the subsequent decoding process.

Let the input be $X\in R^{H\times W\times C_{0}}$, where $C_{0}=3$, and let the base channel width be *C*. In all experiments, the base channel width is set to C=64. MSCB generates three side-branch features, denoted as $\left\{s_{1},s_{2},s_{3}\right\}$, at the spatial scales {1/2,1/4,1/8}, respectively. Their channel dimensions progressively increase along the hierarchy.

MSCB follows a progressive convolution–downsampling strategy. It first extracts a shallow base representation $X_{0}$ at the original resolution and then generates multi-scale side-branch features through successive pooling and convolutional mappings:

(1) $$X_{0}=D_{0}\left(X\right)\in R^{H\times W\times C},$$

(2) $$s_{1}=D_{1}\left(P\left(X_{0}\right)\right)\in R^{\frac{H}{2}\times \frac{W}{2}\times 2C},$$

(3) $$s_{2}=D_{2}\left(P\left(s_{1}\right)\right)\in R^{\frac{H}{4}\times \frac{W}{4}\times 4C},$$

(4) $$s_{3}=D_{3}\left(P\left(s_{2}\right)\right)\in R^{\frac{H}{8}\times \frac{W}{8}\times 8C},$$

where P(·) denotes a 2×2 max-pooling operation, and $D_{i}\left(\cdot \right)$ denotes the local convolutional mapping at the *i*-th stage. Through progressive resolution reduction and channel expansion, MSCB establishes an auxiliary feature hierarchy whose spatial scales correspond to the backbone levels used during decoding.

Each stage employs a standard convolution–normalization–activation block. Since these operations follow conventional implementations, their internal details are omitted for brevity. By construction, $s_{1}$, $s_{2}$, and $s_{3}$ match the spatial resolutions of the corresponding backbone levels used during decoding. The effective output of MSCB is therefore defined as

(5) $$S_{\mathrm{MSCB}}=\left\{s_{1},s_{2},s_{3}\right\}.$$

MSCB is responsible for constructing the input-derived auxiliary feature hierarchy, while the subsequent interaction between these complementary features and the backbone representations is described separately in the feature-fusion stage.

### 3.3. SFM Module

**Figure 4:**
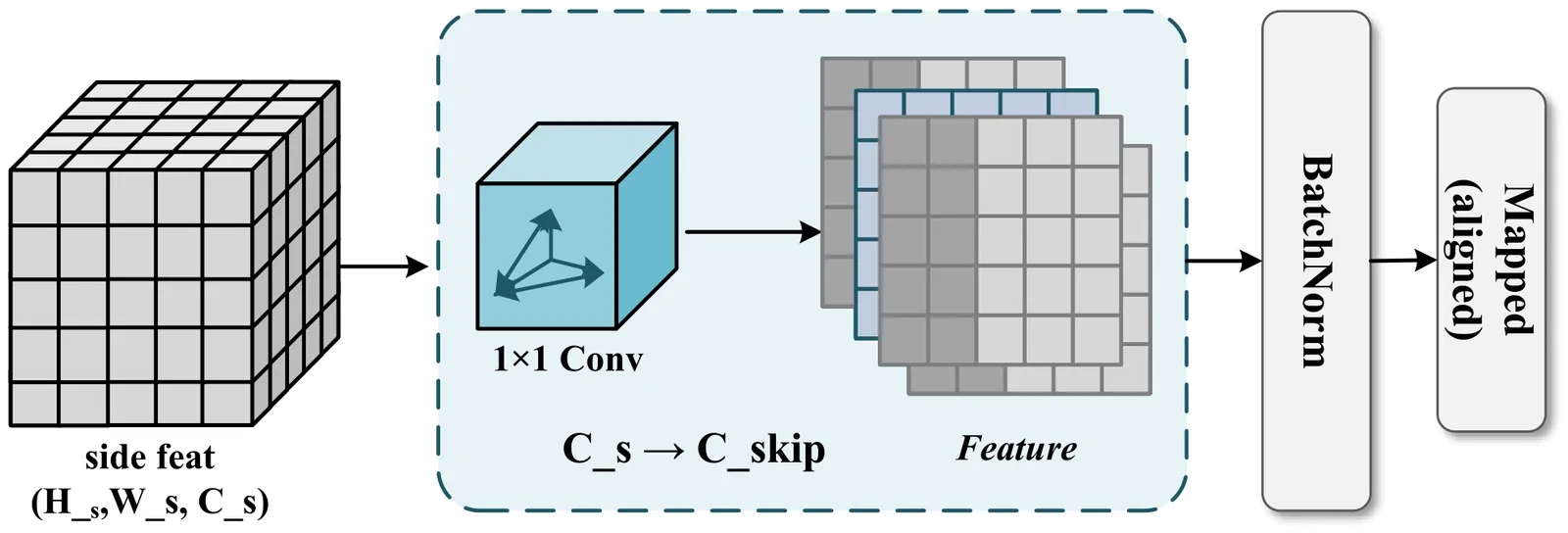
Scale-Consistent Feature Embedding Module (SFM). Side features are projected via a 1 × 1 convolution and normalized to align channel dimensions and statistical distributions before fusion with backbone skip features.

In multi-branch architectures, side-branch features and backbone features are generated through different representation pathways and may therefore differ in their channel responses and statistical characteristics. Directly combining these heterogeneous representations may introduce feature redundancy and semantic interference. Therefore, as illustrated in Figure 4, the Scale-Consistent Feature Embedding Module (SFM) adapts the side-branch representations before residual injection.

Let $F_{s}^{k}$ and $F_{skip}^{k}$ denote the side-branch feature and the corresponding backbone skip feature at level *k*, respectively. SFM adapts and injects the side-branch feature as

(6) $${\hat{F}}_{s}^{k}=BN\left(W_{k}^{1\times 1}*F_{s}^{k}\right),\;F_{skip}^{k\prime }=F_{skip}^{k}+{\hat{F}}_{s}^{k},$$

where $W_{k}^{1\times 1}$ maps the side-branch feature to the channel space of the corresponding skip feature, and BN(·) adjusts its statistical representation. Spatial resizing is applied only when the feature resolutions are inconsistent. The adapted feature is then introduced through residual addition, avoiding the channel expansion and additional redundancy associated with direct concatenation.

The purpose of SFM is not to introduce a new nonlinear feature extractor but to provide a consistent interface between the independently generated MSCB features and the backbone skip representations. It therefore separates complementary information generation from cross-path representation adaptation.

### 3.4. DCM Module

**Figure 5:**
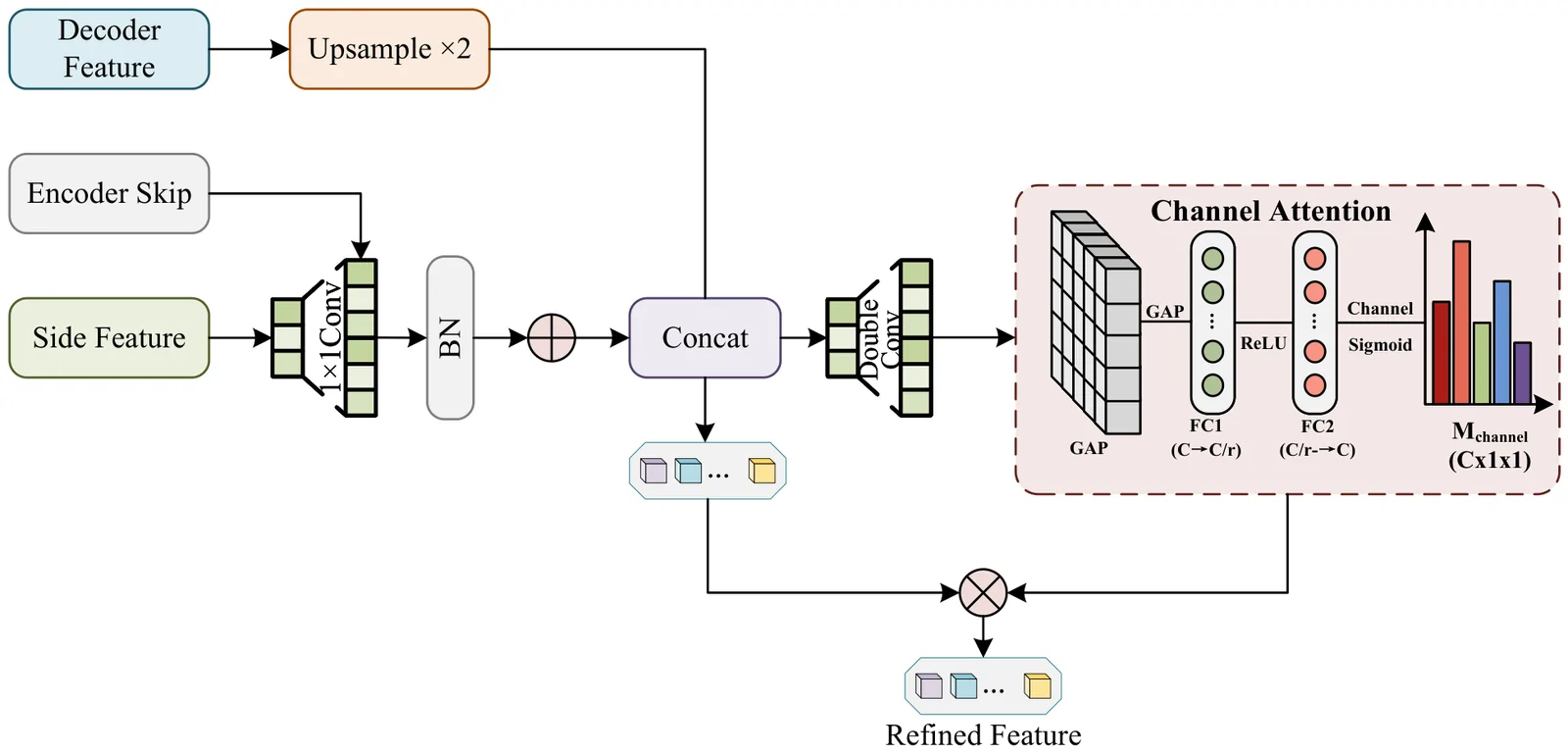
Discriminative Channel Modulation (DCM) module. Fused features are adaptively recalibrated through global context aggregation and channel-wise modulation to enhance discriminative responses before final decoding.

After cross-path feature injection and decoder fusion, task-relevant responses and redundant activations may coexist in the channel dimension of the fused representation. Such redundancy can weaken category discrimination, particularly in complex remote sensing scenes containing small objects and spectrally similar land-cover classes. To regulate the fused representation, a Discriminative Channel Modulation module (DCM) is placed after each decoding fusion stage. As illustrated in Figure 5, DCM generates channel-wise modulation weights from the current decoding feature and adaptively recalibrates its channel responses.

Let $F_{d}\in R^{C\times H\times W}$ denote the feature produced by a decoding unit after skip-feature fusion. DCM performs channel modulation as

(7) $$a=\sigma \left(W_{2}\;\delta \left(W_{1}\;GAP\left(F_{d}\right)\right)\right),\;{\hat{F}}_{d}=a⊙F_{d},$$

where GAP(·) denotes global average pooling, δ(·) is the ReLU activation, σ(·) is the Sigmoid function, and ⊙ denotes channel-wise multiplication. The two transformations use a reduction ratio *r* to generate compact channel descriptors. The reduction ratio is set to r=4 in all experiments.

Rather than serving as an independent feature-extraction pathway, DCM is positioned after decoding-feature fusion to recalibrate the currently available representation. It can therefore modulate either backbone-only decoding features or features containing the complementary information injected by MSCB and SFM. Its role is to strengthen task-relevant channel responses and reduce redundant activations before the next reconstruction stage.

### 3.5. Loss Function

To ensure stable optimization under class-imbalance conditions, this article adopts a combined loss function consisting of weighted cross-entropy and Lovász-Softmax.

The overall loss is defined as

(8) $$L=L_{CE}+L_{Lovasz}$$

(1) Weighted Cross-Entropy Loss

Let the predicted probability be denoted as

$$P_{i,c}=Softmax{\left(y_{i}\right)}_{c}$$

The weighted cross-entropy loss is then defined as

(9) $$L_{CE}=-\sum_{i}\sum_{c}w_{c}\;y_{i,c}\;log\left(p_{i,c}\right)$$

where $w_{c}$ denotes the class weight, which is used to alleviate the class distribution imbalance problem.

(2) Lovász-Softmax Loss

To directly optimize the Intersection over Union (IoU) metric, the Lovász-Softmax loss is introduced:

(10) $$L_{Lovasz}=\frac{1}{K}\sum_{c=1}^{K}\bar{\Delta_{Jaccard}}\left(m_{c}\right)$$

This loss approximates the convex surrogate of the IoU for each class, thereby improving the consistency of segmented regions.

(3) Joint Optimization Objective

The final training objective is defined as

(11) $$\min_{\theta }E_{\left(X,Y\right)}\left[L_{CE}+L_{Lovasz}\right]$$

The combined loss simultaneously considers pixel-level classification accuracy and region-level structural consistency during model optimization.

## 4. Experiment

### 4.1. Dataset

High-resolution remote sensing images are typically defined as those with a ground sampling distance (GSD) of less than 1 m. To evaluate the applicability and robustness of the proposed method under different annotation schemes and scene complexities, experiments were conducted on two remote sensing datasets: the ISPRS Vaihingen Dataset and a self-annotated dataset. The Vaihingen dataset provides clearly defined semantic categories and is suitable for evaluating the capability of segmentation models in performing fine-grained urban land-cover segmentation. It contains 33 true orthophoto (TOP) images captured by advanced airborne sensors, covering an area of approximately 1.38 km^2^ in the Vaihingen region. The self-annotated dataset is constructed based on high-resolution orthorectified remote sensing imagery. The images were manually annotated and categorized using ArcGISPro, and the final labels were exported as raster images. Compared with the Vaihingen dataset, the self-annotated dataset adopts a semantic category definition that is closer to practical application scenarios. The semantic similarity among categories is higher, and spatial mixing between classes is more severe, which imposes higher requirements on the model’s high-level semantic representation capability and robustness. By evaluating the proposed method on these two datasets with significantly different yet complementary annotation styles, the generalization capability and practical applicability of the proposed framework can be comprehensively assessed.

#### 4.1.1. ISPRS Vaihingen Dataset

The ISPRS Vaihingen Dataset [72] consists of 33 true orthophoto (TOP) images captured by advanced airborne sensors, covering an area of approximately 1.38 km^2^ in the Vaihingen region. The ground sampling distance (GSD) of these images is approximately 0.09 m. Each TOP image contains three channels: infrared (IR), red (R), and green (G).

**Figure 6:**
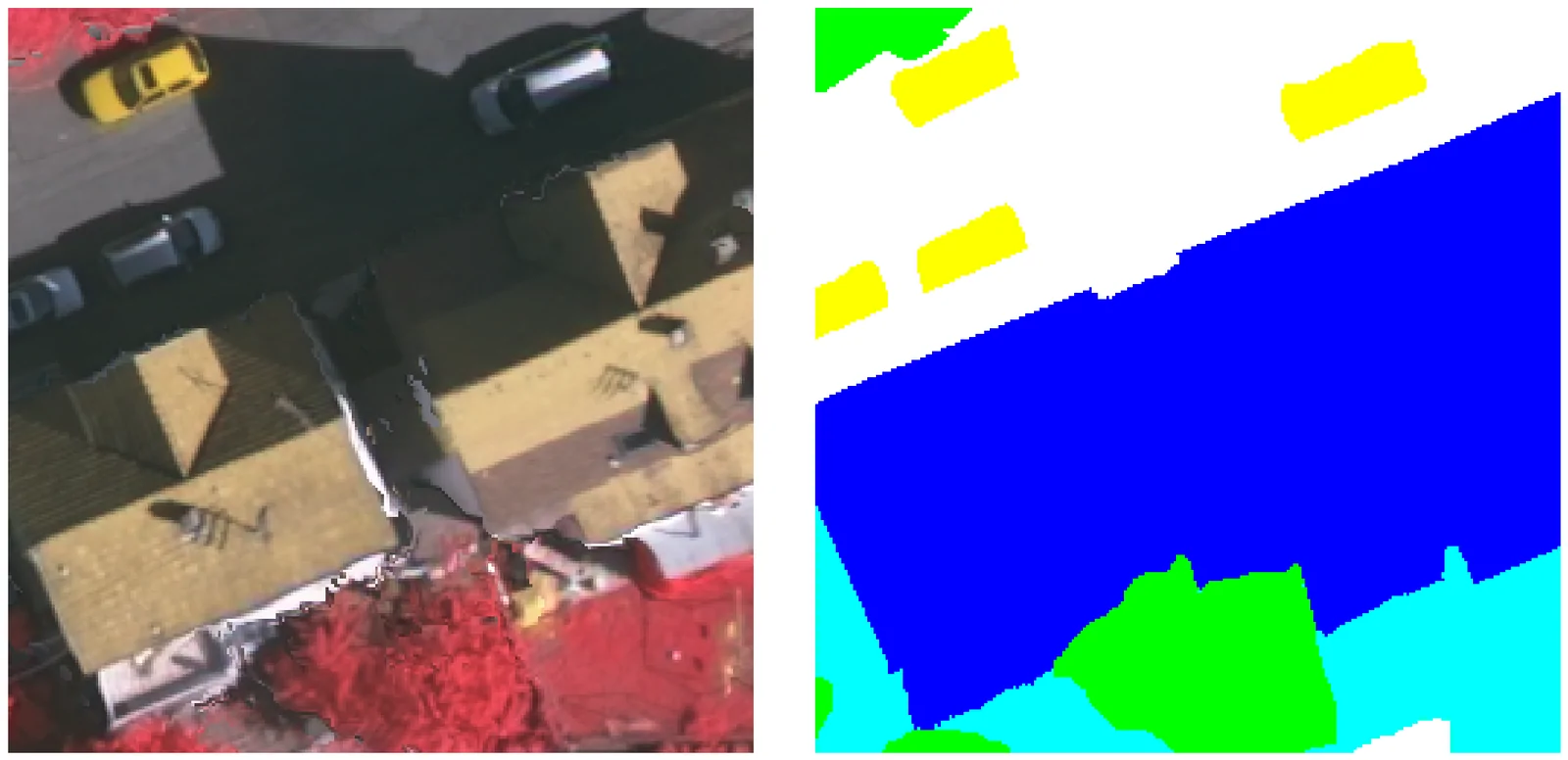
The IRRG and GT of a sample in the ISPRS Vaihingen dataset.

The dataset provides pixel-level annotations for six land-cover categories: impervious surfaces (white), low vegetation (cyan), buildings (blue), cars (yellow), trees (green), and background (red). Following [37,57,73,74], the images with IDs 1, 3, 5, 7, 13, 17, 21, 23, 26, 32, and 37 are used for training, while the images with IDs 11, 15, 28, 30, and 34 are used for testing. All images were cropped into patches of size 256 × 256. To avoid the loss of image information near the boundaries during sample generation and to ensure a consistent input size, a Boundary-Adaptive Cropping strategy was adopted. Specifically, a sliding window moves across the image with a fixed stride *s*, where s=patch_size−overlap. For regions near the right and bottom boundaries, when the remaining image extent is insufficient for a complete window movement, zero padding is not applied. Instead, the starting coordinates of the window are dynamically adjusted so that its right and bottom edges are strictly aligned with the boundaries of the original image:

(12) x=min(j×s,W−w),y=min(i×s,H−h),

where (x,y) denotes the coordinates of the upper-left corner of the cropped patch, (W,H) represents the width and height of the original image, and (w,h) denotes the size of the cropped patch. This strategy ensures full coverage of the original image while guaranteeing that each generated sample contains only valid geographic information, thereby avoiding padding-induced artifacts that may interfere with network feature extraction. Figure 6 illustrates example samples from the Vaihingen dataset.

#### 4.1.2. Self-Annotated Dataset

**Figure 7:**
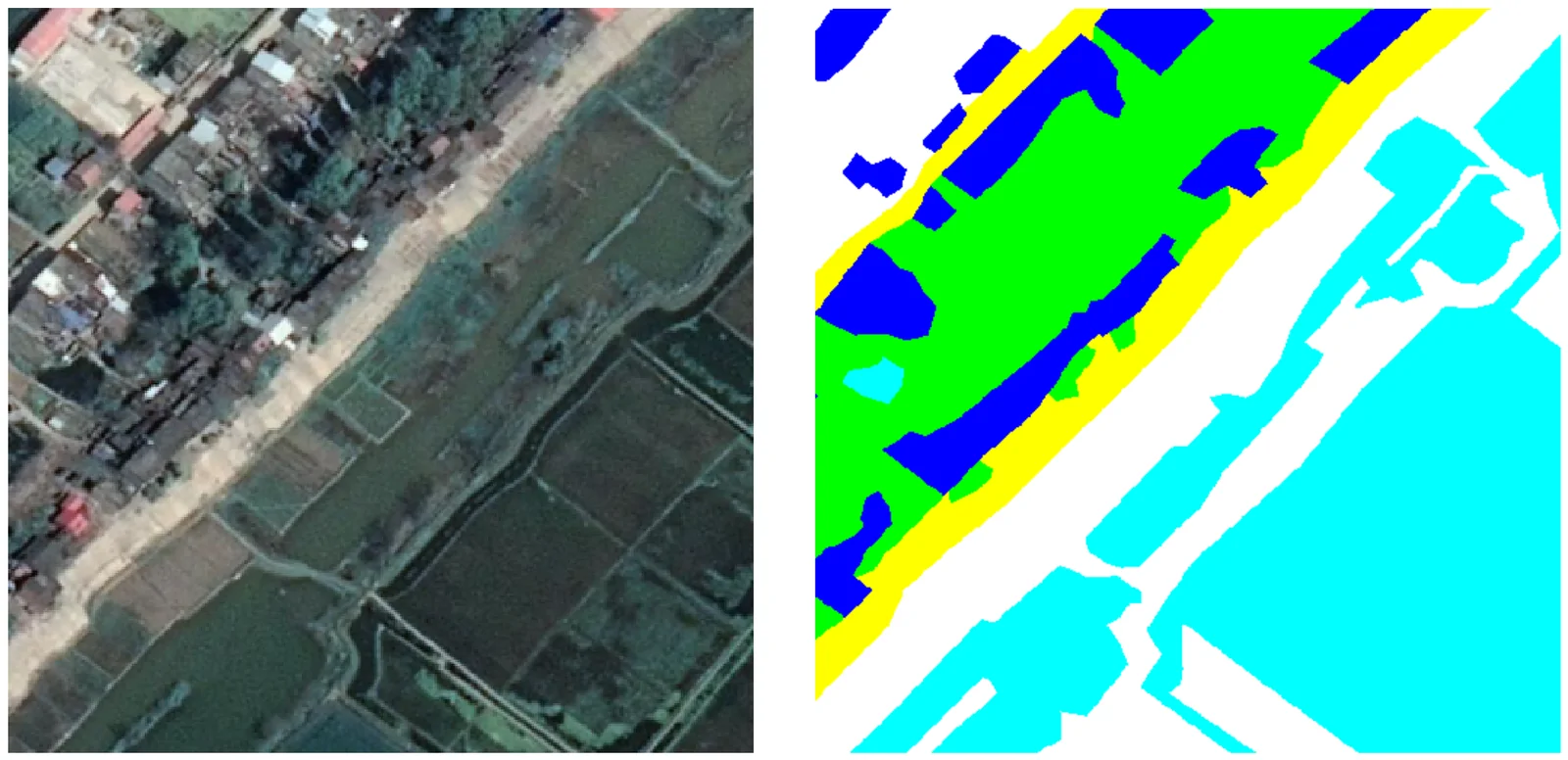
The image and GT of a sample in the self-annotated dataset.

The self-annotated dataset was collected from Yueyang. The land-cover annotations in this dataset include five categories: buildings, roads, water bodies, trees, and background. The ground sampling distance (GSD) of these images is approximately 0.53 m. Before annotation, the source images were standardized in terms of file format, channel configuration, and spatial resolution. Pixel-level annotations were manually delineated in ArcGIS Pro using polygon editing tools and were subsequently rasterized into single-channel semantic masks. Annotation quality was controlled by overlaying the vector polygons and rasterized masks on the original images to identify omitted regions, incorrect category assignments, discontinuous annotations, and image–label misalignment, followed by programmatic verification of image–label correspondence, spatial dimensions, and label values. The original remote sensing imagery and the corresponding annotation maps were first partitioned into spatially disjoint training and test regions. The imagery and labels within the two regions were then separately cropped into non-overlapping patches of 512 × 512 pixels while maintaining spatial consistency between the images and annotations. After patch extraction, the resulting training and test samples were approximately distributed at a 9:1 ratio. Figure 7 presents example samples from the self-annotated dataset.

**Figure 8:**
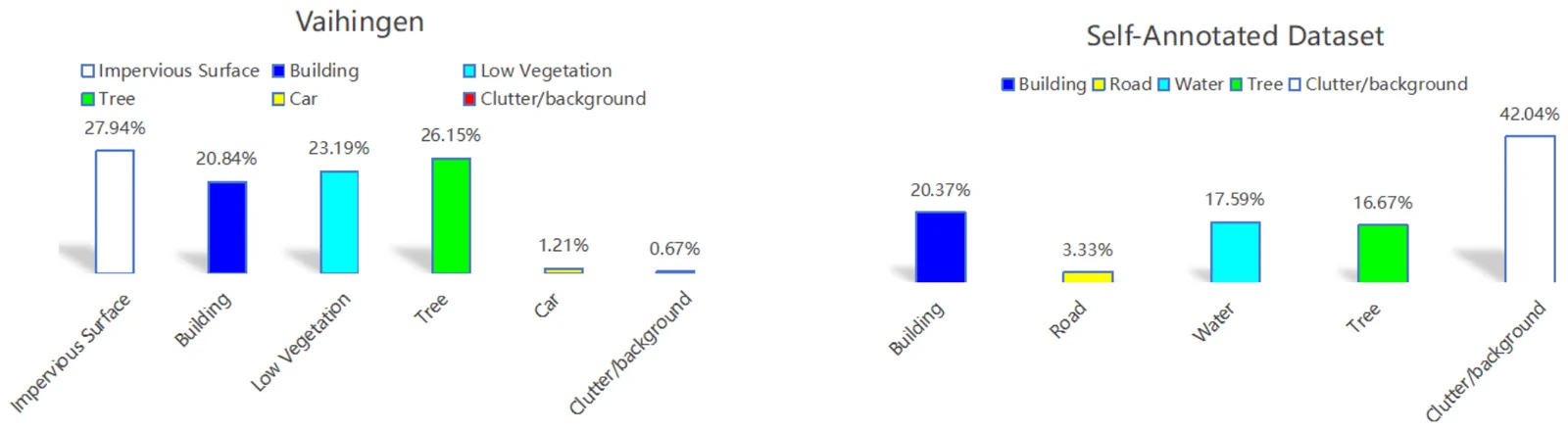
Proportion of each semantic label in the Vaihingen and self-annotated datasets.

Figure 8 illustrates the proportion of each semantic category in the two aforementioned datasets. The class distribution of the self-annotated dataset is highly imbalanced. As shown in Figure 8, Road accounts for only approximately 3.33% of all pixels, whereas Building, Water, and Tree account for approximately 20.37%, 17.80%, and 18.46%, respectively. Therefore, Road constitutes an extremely sparse category in the dataset. In addition, its narrow and elongated geometry makes its effective spatial support substantially smaller than that of the other foreground classes. Following the experimental protocols adopted in [52,75], the background category is ignored during the quantitative evaluation on the ISPRS Vaihingen Dataset. Similarly, the background category is also excluded from the evaluation of the self-annotated dataset.

### 4.2. Experimental Setup

#### Training Configuration and Comparative SOTA Methodology

This experiment is implemented using the open-source framework PyTorch 2.1.0. The proposed SEMU-Net model is trained for 120 epochs, with a total parameter size of approximately 50.41 M. The AdamW optimizer is adopted with a weight decay of $1\times 10^{-4}$. The initial learning rate is set to $1\times 10^{-4}$, and a cosine annealing learning rate schedule is employed to gradually decay the learning rate to $1\times 10^{-6}$. The training batch size is set to 8, and the validation batch size is set to 1. All models are trained on an NVIDIA GeForce RTX 3090 24 GB GPU, with automatic mixed precision (AMP) enabled to accelerate training. The baseline model adopts U-Net, and the loss function is a combination of weighted cross-entropy and Lovász-Softmax. The available training samples were further divided into training and validation subsets at a ratio of 9:1. The checkpoint with the highest validation mIoU was selected for the final evaluation; when the validation mIoU was identical, the checkpoint with the higher Average F1 was retained. To ensure the stability and reliability of the experimental results, five independent runs were conducted for each model in the main comparative experiments under the same experimental configuration. The mIoU and Average F1 results are reported as the mean ± standard deviation obtained from the five runs.

To verify the effectiveness and competitiveness of the proposed method, the network is compared with several representative semantic segmentation methods on the Vaihingen dataset. These include CNN-based models such as U-Net, PSPNet, and DeepLabV3+. Transformer-based or hybrid architectures are also considered, including UNetFormer and A^2^-FPN. The CMT model connects CNN and Transformer structures in a serial manner, while SCG-TransNet adopts a Transformer as the primary encoder for feature extraction. ST-UNet employs a parallel architecture that integrates Transformer and CNN branches for feature representation. ESMS-Net consists of PPAFormer, DFFM, MFWF, and an auxiliary enhanced encoder to achieve collaborative multi-module modeling. In addition, the structurally optimized AFENet is also included in the experimental comparison.

To evaluate segmentation performance, three metrics are adopted: Mean Intersection over Union (MIoU), and mean F1-score. These metrics are computed based on the confusion matrix, which includes four terms: True Positive (TP), False Positive (FP), True Negative (TN), and False Negative (FN). The formulas for OA, F1-score, and Intersection over Union (IoU) are defined as follows:

(13) $$\mathrm{IoU}=\frac{\mathrm{TP}}{\mathrm{TP}+\mathrm{FP}+\mathrm{FN}}$$

(14) $$F1=2\times \frac{\mathrm{precision}\times \mathrm{recall}}{\mathrm{precision}+\mathrm{recall}}$$

where Precision =TP/(TP+FP) and Recall =TP/(TP+FN). For multi-class segmentation tasks, MIoU denotes the average IoU across all classes, while mean F1 represents the average F1-score over all categories.

### 4.3. Experimental Results

To systematically evaluate the performance of the proposed SEMU-Net for land-cover segmentation in high-resolution remote sensing imagery, comparative experiments were conducted on the public ISPRS Vaihingen Dataset as well as a self-constructed high-resolution land-cover dataset.The comparison methods include UNet, PSPNet, Deeplabv3+, UNetFormer, CMT, A^2^-FPN, ST-UNet, SCG-TransNet, ESMS-Net, and AFENet. These comparison methods were selected to ensure the comprehensiveness and fairness of the evaluation.

#### 4.3.1. Experimental Results on the ISPRS Vaihingen Dataset

Table 1 presents the comparison results of class-wise Intersection over Union (IoU) and overall evaluation metrics for different methods on the Vaihingen dataset. It can be observed that the proposed SEMU-Net achieves competitive segmentation performance across multiple key categories.

For the typical structural land-cover category Building, SEMU-Net achieves a segmentation accuracy of 84.00% IoU, representing an improvement of approximately 1.97% compared with the traditional U-Net (82.03%). This result indicates that the parallel side-path compensation branch, which independently models shallow spatial texture information, can effectively alleviate the progressive degradation of structural information caused by successive downsampling in the main pathway, thereby enhancing the representation capability of deep features for regularly shaped objects.

For the Car category, which exhibits significant scale variation and relatively small object sizes, SEMU-Net achieves the best performance with 67.53% IoU, outperforming the recently competitive AFENet (64.03%) by approximately 3.50%. This result indicates that the proposed framework performs favorably in small-object segmentation, and the observed improvement is consistent with the design objectives of multi-scale complementary information and cross-path feature adaptation. The sensitivity of small vehicles to scale variation and complex background interference has also been reported in aerial vehicle analysis [8].

In terms of overall evaluation metrics, SEMU-Net achieves mIoU = 72.21% and Average F1 = 83.64%. Compared with the high-performing models ESMS-Net (2024) and AFENet (2025), SEMU-Net improves the mIoU by approximately 1.68 and 0.59 percentage points, respectively. These results indicate that the coordinated design of complementary feature construction, cross-path adaptation, and channel recalibration contributes to improved overall segmentation performance.

**Figure 9:**
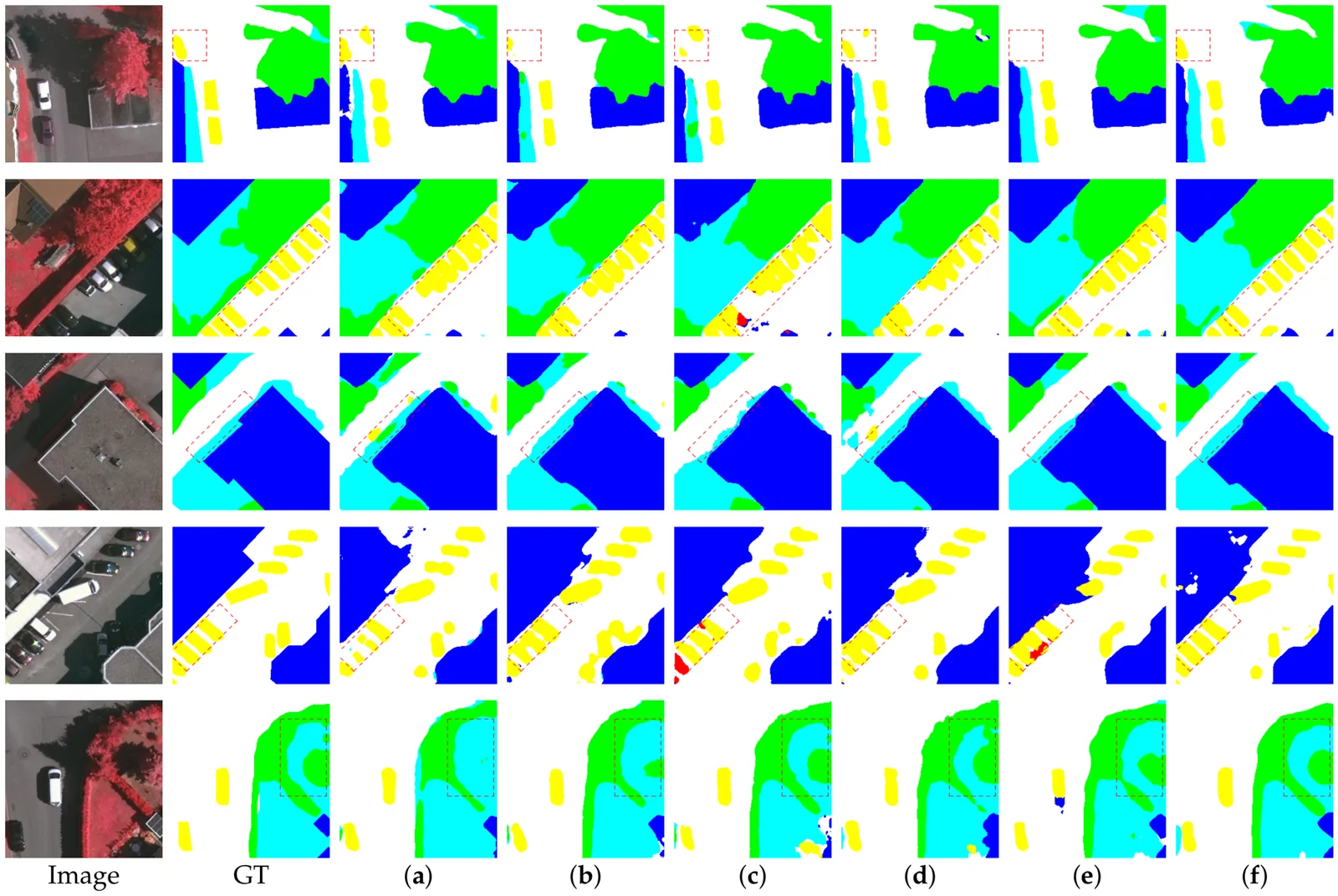
Visualization results of selected networks on the Vaihingen dataset. (**a**) UNet. (**b**) Deeplab V3+. (**c**) CMT. (**d**) ESMS-Net. (**e**) AFENet. (**f**) ours.

**Figure 10:**
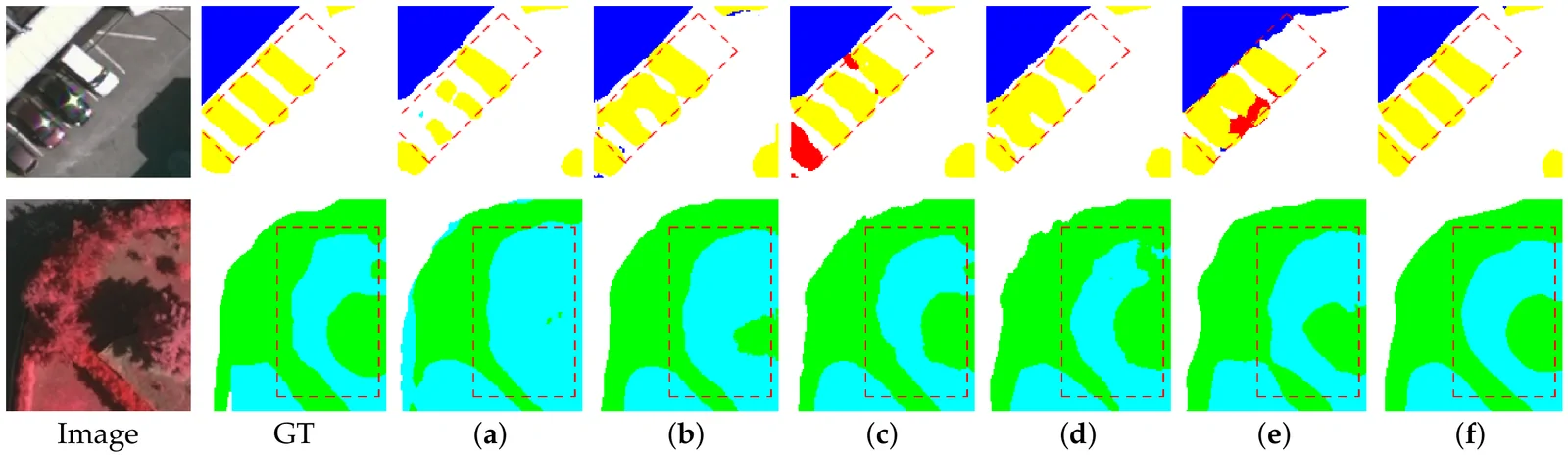
Enlarged qualitative comparison of representative challenging regions selected from the fourth and fifth rows of Figure 9. Identical regions are cropped from the input images, ground-truth maps, and predictions of all compared methods to facilitate local comparison. (**a**) UNet. (**b**) Deeplab V3+. (**c**) CMT. (**d**) ESMS-Net. (**e**) AFENet. (**f**) ours.

To provide a visual comparison, Figure 9 presents representative segmentation results on the Vaihingen test set, while Figure 10 provides enlarged views of selected local regions.

#### 4.3.2. Results on the Self-Annotated Dataset

To further evaluate the performance of SEMU-Net under a different class distribution and image source, comparative experiments were conducted on the self-annotated land-cover dataset. The quantitative results are presented in Table 2.

From the class-wise results, SEMU-Net achieves the highest IoUs for Road and Tree, reaching 57.41% and 80.08%, respectively. Its Road IoU is 4.12 percentage points higher than that of AFENet. However, Road remains the category with the lowest absolute IoU. This is mainly because roads account for only approximately 3.33% of the dataset pixels and generally exhibit narrow, elongated, and branching spatial patterns. Small localization errors, local discontinuities, vegetation occlusion, shadows, and visual similarity to surrounding surfaces therefore have a relatively large influence on the Road IoU.

In terms of overall performance, SEMU-Net achieves a mean mIoU of 76.11% and a mean Average F1 of 85.94%. These values are 1.36 and 1.08 percentage points higher than those of AFENet, respectively. Although SEMU-Net does not obtain the highest Building and Water IoUs, its improvements on Road and Tree contribute to the best overall performance among the compared methods.

**Figure 11:**
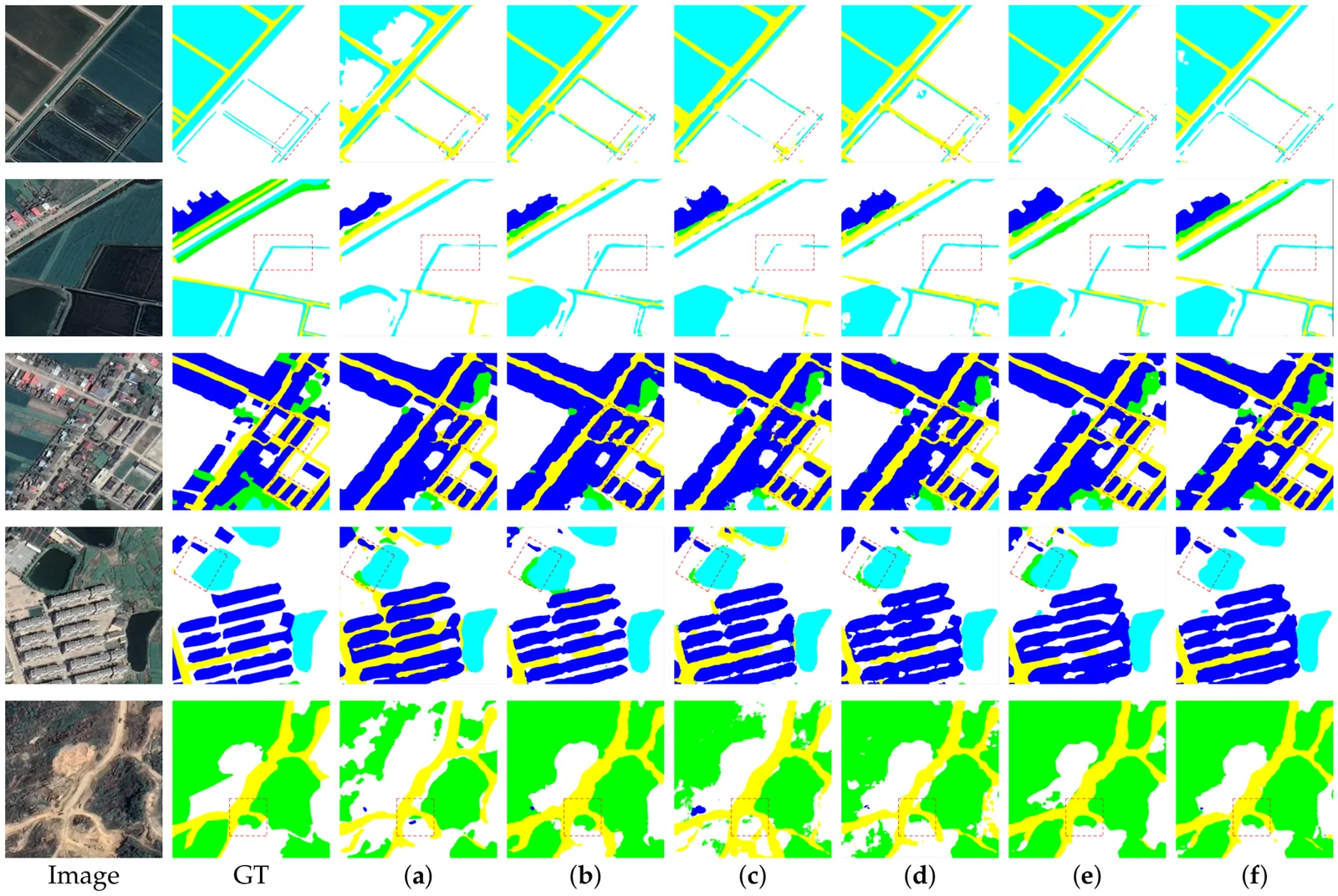
Visualization results of selected networks on the self-annotated dataset. (**a**) UNet. (**b**) Deeplab V3+. (**c**) CMT. (**d**) ESMS-Net. (**e**) AFENet. (**f**) ours.

**Figure 12:**
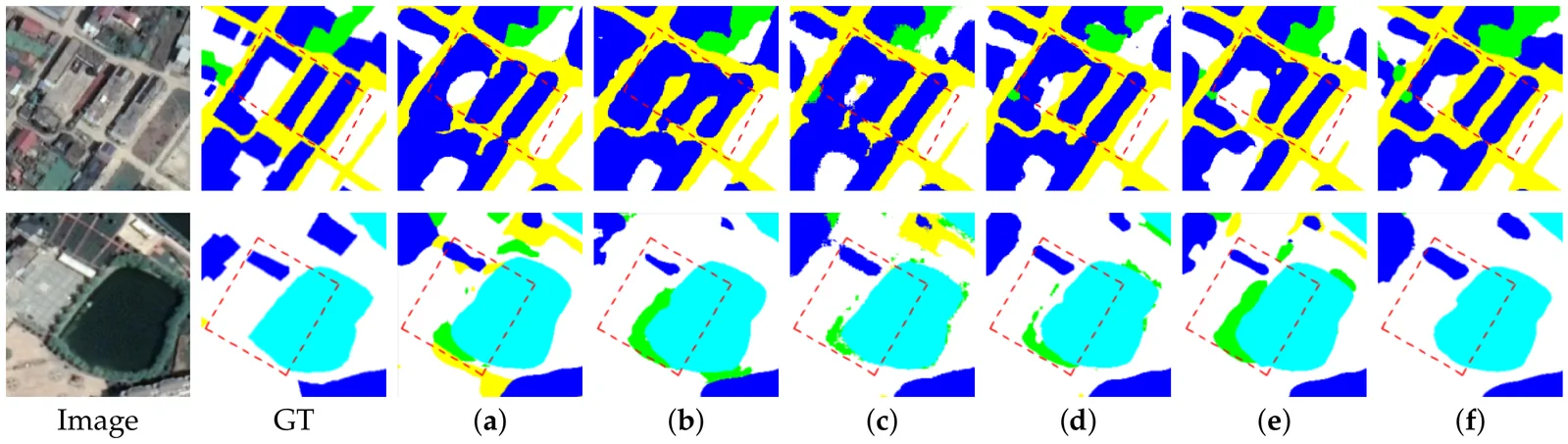
Enlarged qualitative comparison of representative regions selected from the third and fourth rows of Figure 11. Identical spatial regions are cropped from the input images, ground-truth maps, and predictions of all compared methods to facilitate local comparison. (**a**) UNet. (**b**) Deeplab V3+. (**c**) CMT. (**d**) ESMS-Net. (**e**) AFENet. (**f**) ours.

Figure 11 presents representative segmentation results on the self-annotated test set, and Figure 12 provides enlarged views of selected local regions for a more detailed comparison.

SEMU-Net achieves the highest mean mIoU and Average F1 among the compared methods, while its category-level gains are mainly observed for Road and Tree rather than uniformly across all classes. This indicates that the improvement is category dependent rather than uniform across all land-cover classes.

## 5. Discussion

### 5.1. Ablation Study

**Figure 13:**
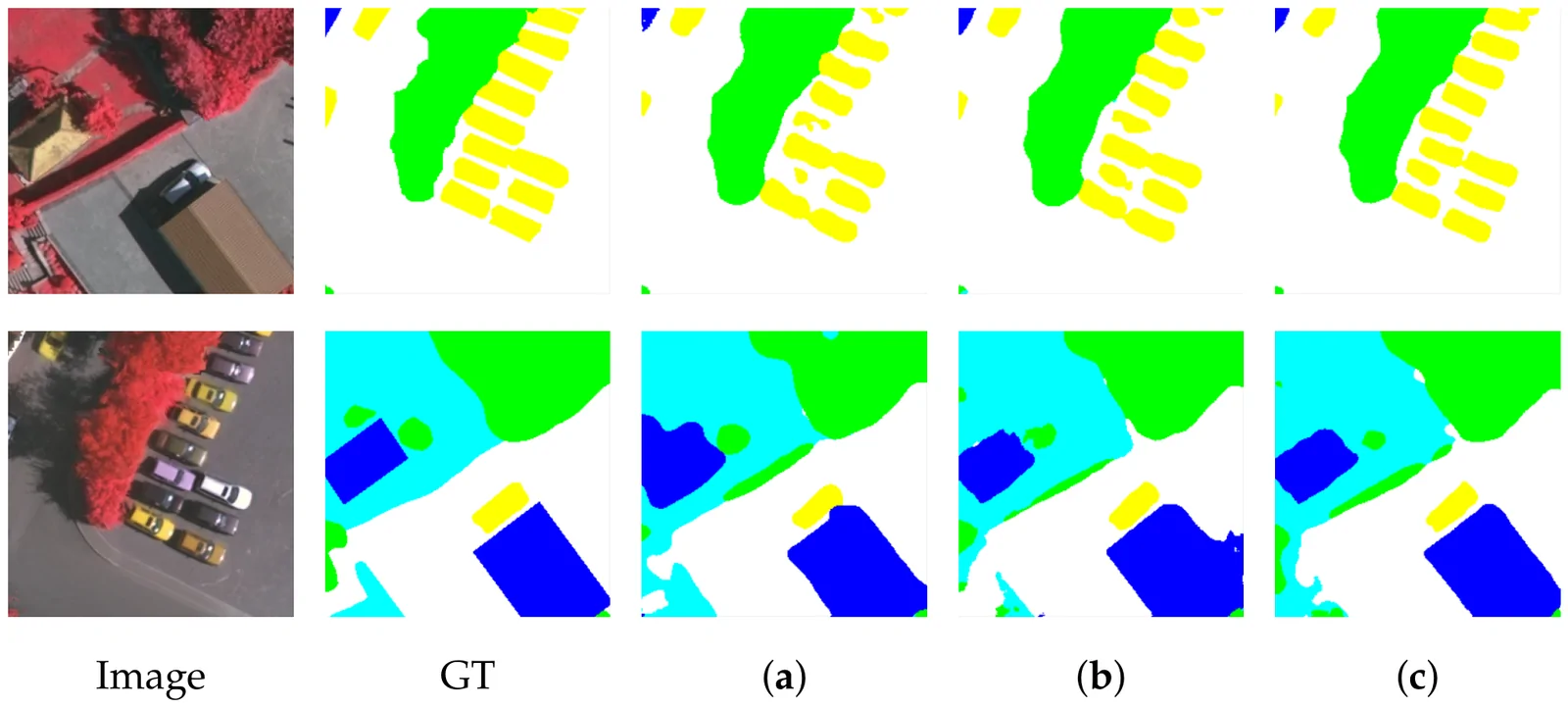
Visualization results of the ablation study on the Vaihingen dataset. (**a**) w/o MSCB and SFM. (**b**) w/o DCM. (**c**) Ours.

To evaluate the individual and collaborative contributions of DCM, MSCB, and SFM, module-removal ablation experiments were conducted on the Vaihingen dataset using U-Net as the baseline. Only structurally valid module combinations were considered, and the quantitative results are reported in Table 3. Figure 13 presents visualization results of ablation studies on the Vaihingen dataset. Before discussing the quantitative results, the dependency relationships among the three modules are first clarified.

MSCB, SFM, and DCM do not form a fully serial dependency. SFM is specifically designed to adapt the side-branch features generated by MSCB before their injection into the backbone decoding pathway. Therefore, SFM cannot operate independently when MSCB is removed, and no configuration retaining SFM without MSCB is considered. In contrast, DCM performs channel recalibration on the single feature tensor available at the current decoding stage and does not require MSCB features as an independent input. In the complete model, this tensor contains the side-branch information adapted and injected through SFM. When MSCB and SFM are removed, it reduces to the decoding feature produced by the original U-Net pathway, allowing DCM to operate directly on the backbone feature. Accordingly, the “w/o MSCB & SFM (DCM only)” configuration is equivalent to U-Net equipped only with DCM and is used to evaluate the independent contribution of DCM. On the basis of these structural relationships, the effects of the individual modules are analyzed first.

Compared with the U-Net baseline, which achieves a mIoU of 67.72%, retaining only DCM increases the mIoU to 69.18%, corresponding to an improvement of 1.46 percentage points. Retaining MSCB with direct fusion yields a mIoU of 68.87%, improving the baseline by 1.15 percentage points. These results show that both DCM and MSCB can independently improve the baseline representation. However, the moderate gain obtained by MSCB alone also indicates that introducing an auxiliary pathway does not automatically ensure effective utilization of the complementary information. This observation further motivates an examination of the role of SFM in adapting the side-branch features before fusion.

When SFM is introduced together with MSCB but without DCM, the mIoU increases from 68.87% to 69.73%, corresponding to a conditional gain of 0.86 percentage points. A similar trend is observed when DCM is also retained: introducing SFM further improves the mIoU from 70.89% to 72.21%. These comparisons indicate that SFM contributes only when MSCB is active and that adapting the side-branch features before fusion is more effective than direct element-wise addition. Nevertheless, the current evidence is based on segmentation performance and does not directly quantify whether SFM reduces the statistical discrepancy between the two feature pathways. Having established the conditional contribution of SFM, the role of DCM within different feature configurations is examined next.

Removing DCM from the complete model reduces the mIoU from 72.21% to 69.73%. In contrast, DCM alone improves the U-Net baseline by 1.46 percentage points. Taken together, these results suggest that DCM can independently recalibrate the original decoding features and provides a larger contribution when applied to representations containing the complementary information introduced by MSCB and SFM. However, because its channel weights are generated from globally pooled descriptors, spatially varying responses are compressed into a single channel representation, limiting its ability to explicitly distinguish localized ambiguities. The above module-level comparisons collectively explain the performance of the complete configuration.

Overall, the complete model achieves the best performance, with a mIoU of 72.21% and an Average F1 of 83.64%. Compared with the U-Net baseline, the Car and Impervious Surfaces IoUs increase by 13.52 and 3.73 percentage points, respectively. The category-level gains are not uniform, with larger improvements observed for categories that rely strongly on fine-grained spatial cues, while class imbalance, spectral similarity, intra-class variation, and the connectivity of elongated objects remain challenging. In addition, the additional feature-extraction pathway introduced by MSCB contributes to the increased parameter count and computational cost. Therefore, the ablation results support the independent effectiveness of MSCB and DCM and the conditional contribution of SFM, while also revealing the computational and representation limitations of the current design.

To further examine whether the performance improvement of SEMU-Net primarily results from its increased model capacity, an additional capacity-controlled experiment was conducted. Specifically, the channel width of the original U-Net was increased while its encoder–decoder topology, skip connections, training strategy, and loss function were kept unchanged, resulting in a parameter count comparable to that of SEMU-Net.

As shown in Table 4, increasing the capacity of U-Net from 22.70 M to 50.08 M parameters improves the mIoU from 67.72% to 69.14%, indicating that increased model capacity itself contributes to segmentation performance. However, despite having a comparable parameter count, the capacity-matched U-Net remains 3.07 percentage points lower in mIoU and 2.38 percentage points lower in Average F1 than SEMU-Net. This comparison indicates that the performance improvement of SEMU-Net cannot be explained solely by the increase in parameter capacity and provides additional support for the contribution of its coordinated multi-branch architecture. Nevertheless, SEMU-Net requires substantially more FLOPs, and its computational overhead remains an important limitation.

### 5.2. Accuracy–Complexity Trade-Off

As shown in Table 5, SEMU-Net achieves the highest segmentation accuracy among the compared methods, but this performance is obtained with a relatively large computational budget. The model contains 50.41 M parameters and requires 72.67 GFLOPs, compared with 20.23 M parameters and 12.50 GFLOPs for AFENet. Thus, SEMU-Net uses approximately 2.49 times as many parameters and 5.81 times as many floating-point operations, while its mIoU and Average F1 are higher by 0.59 and 0.44 percentage points, respectively. This comparison places SEMU-Net toward the accuracy-oriented end of the accuracy–complexity spectrum rather than among lightweight segmentation architectures.

The additional complexity mainly arises from the independent multi-scale complementary branch and the repeated feature processing performed during decoding. These components provide auxiliary representations for fine-grained semantic reconstruction, but they also increase model size, intermediate feature storage, and computational demand. Accordingly, the practical suitability of SEMU-Net depends on the target application. It is more appropriate for offline or workstation-based remote sensing analysis, where segmentation accuracy is prioritized and sufficient computational resources are available. In contrast, lightweight networks such as AFENet may be preferable for embedded, edge, or real-time applications that impose stricter constraints on memory, latency, and throughput.

Parameter count and GFLOPs provide architecture-level measures of model complexity, but they do not fully determine actual deployment performance. Inference latency, memory consumption, throughput, and energy efficiency also depend on hardware architecture, operator implementation, memory-access patterns, and software optimization. Since hardware-level latency and energy measurements are not included in the present study, the above deployment analysis should be interpreted as an assessment based on computational complexity rather than direct runtime evidence.

Recent studies have investigated weight pruning, parameter quantization, and lightweight multi-scale modeling to reduce deployment costs [76]. Although these techniques were originally developed for a different recognition task, they provide relevant directions for improving the efficiency of SEMU-Net. Potential strategies include reducing the channel widths of MSCB, sharing parameters across its stages, replacing standard convolutions with depthwise separable or other efficient operators, and applying structured channel pruning or low-bit quantization after training. Future work will evaluate these strategies together with inference latency, peak memory usage, throughput, and energy consumption on representative GPU and edge platforms.

### 5.3. Limitations and Future Improvements

Despite the observed improvements, SEMU-Net has several limitations. First, the independent side branch and repeated decoder modulation substantially increase the parameter count and computational complexity. This limits direct deployment in resource-constrained or real-time remote sensing applications.

Second, SFM is evaluated indirectly through segmentation performance. Although the ablation results support the practical value of channel projection and normalization, the current study does not provide a direct measurement of cross-path feature-distribution discrepancy. Similarly, DCM is evaluated through output performance rather than explicit visualization or quantitative analysis of channel responses. Therefore, the mechanism interpretations presented above should be understood as being supported by structural design and conditional ablation behavior rather than as direct measurements of internal representations.

Third, the current framework does not contain explicit topology- or boundary-specific supervision. This limitation is particularly relevant to the Road class in the self-annotated dataset, whose absolute IoU remains relatively low despite outperforming the competing methods. Severe pixel imbalance, narrow geometry, partial occlusion, and spectral similarity with surrounding surfaces remain challenging for the current pixel- and region-based optimization objectives.

Finally, the experiments are limited to the Vaihingen dataset and one self-annotated dataset. Although these datasets contain different class definitions and scene characteristics, broader cross-region, cross-sensor, and domain-shift experiments are required before stronger generalization claims can be made.

Future work will focus on three aspects. First, lightweight side-branch construction, parameter sharing, pruning, and quantization will be investigated to reduce the computational cost of SEMU-Net. Second, feature-response visualization, quantitative feature-distribution analysis, and channel-weight analysis will be introduced to examine the internal behavior of SFM and DCM more directly. Third, class-balanced sampling, overlapping patch extraction, spatially aware modulation, and topology-aware supervision will be explored to improve the segmentation of elongated and highly imbalanced categories.

## 6. Conclusions

This study proposes SEMU-Net, a structure-enhanced U-shaped network for high-resolution remote sensing image segmentation. The model integrates a Multi-Scale Complementary Branch (MSCB), a Scale-Consistent Feature Mapping module (SFM), and a Discriminative Channel Modulation module (DCM). MSCB constructs an input-derived multi-scale feature hierarchy to supplement fine-grained information weakened during hierarchical downsampling. SFM adapts the side-branch representations through channel projection, normalization, and residual injection before fusion, while DCM recalibrates the fused decoding features to strengthen task-relevant channel responses. Through this coordinated design, SEMU-Net improves the utilization of complementary information during decoding.

Experimental results on the ISPRS Vaihingen dataset and the self-annotated dataset demonstrate the effectiveness of the proposed method under two different category settings. SEMU-Net achieves a mIoU of 72.21% and an Average F1-score of 83.64% on the Vaihingen dataset and a mIoU of 76.11% on the self-annotated dataset. The class-wise results show comparatively pronounced improvements for fine-grained categories such as Car and Road. The ablation experiments further demonstrate the independent contributions of MSCB and DCM, as well as the conditional contribution of SFM when MSCB is active. Overall, the results indicate that coordinating input-derived feature compensation, cross-path feature adaptation, and channel recalibration provides an effective approach to improving high-resolution remote sensing semantic segmentation. Future work will focus on reducing computational complexity, improving the representation of elongated and highly imbalanced categories, and conducting feature-response visualization and channel-weight analysis to examine the internal behavior of the proposed modules more directly.

## Figures and Tables

**Table 1:** Comparison of segmentation results on the Vaihingen Dataset. Bold values indicate the best-performing results, and underlined values represent the second-best performance.

Method	IoU (%)					Evaluation Index	
	Impervious Surfaces	Building	Low Vegetation	Tree	Car	MIoU (%)	Average F1 (%)
UNet	73.25	82.03	57.42	71.89	54.01	67.72±0.39	79.90±0.24
PSPNet	73.84	82.09	56.82	71.73	54.67	67.83±0.28	80.05±0.31
Deeplabv3+	74.95	82.93	56.92	71.91	53.59	68.06±0.34	80.22±0.18
UNetFormer	76.54	81.06	58.81	72.68	54.91	68.80±0.52	81.05±0.37
CMT	77.26	82.38	58.02	71.84	59.28	69.76±0.23	81.78±0.29
A2-FPN	77.12	83.26	58.27	71.95	59.25	69.97±0.31	81.92±0.17
ST-UNet	76.60	82.33	57.87	72.42	61.08	70.06±0.27	82.02±0.34
SCG-TransNet	74.11	82.04	58.10	71.23	63.97	69.89±0.45	81.98±0.26
ESMS-Net	77.43	82.18	58.63	**73.29**	61.11	70.53±0.19	82.45±0.22
AFENet	**77.45**	83.94	59.55	73.11	64.03	$\underline{71.62\pm 0.33}$	$\underline{83.20\pm 0.15}$
Ours	76.98	**84.00**	**59.71**	72.84	**67.53**	72.21±0.29	83.64±0.21

**Table 2:** Quantitative comparison of different segmentation networks on the self-annotated dataset. Bold values indicate the best-performing results, and underlined values represent the second-best performance.

Method	IoU (%)				Evaluation Index	
	Building	Road	Water	Tree	MIoU (%)	Average F1 (%)
UNet	77.64	51.28	85.16	75.36	72.36±0.58	83.41±0.35
PSPNet	76.98	53.46	84.09	73.95	72.12±0.42	83.23±0.49
Deeplabv3+	77.85	53.63	84.91	74.29	72.67±0.51	83.62±0.28
UNetFormer	79.42	52.88	84.76	74.38	72.86±0.36	83.69±0.44
CMT	78.63	52.75	85.97	74.37	72.93±0.57	83.71±0.32
A2-FPN	79.26	52.69	84.96	73.93	72.71±0.29	83.75±0.38
ST-UNet	79.88	53.26	85.22	73.96	73.08±0.47	83.84±0.25
SCG-TransNet	79.14	51.38	86.71	73.05	72.57±0.55	83.31±0.46
ESMS-Net	80.62	53.74	85.68	73.88	73.48±0.34	84.11±0.21
AFENet	**81.23**	53.29	**88.24**	76.24	$\underline{74.75\pm 0.41}$	$\underline{84.86\pm 0.30}$
Ours	79.69	**57.41**	87.25	**80.08**	76.11±0.37	85.94±0.26

**Table 3:** Ablation study on the Vaihingen dataset.

Module	Modules			IoU (%)					Metrics	
	DCM	MSCB	SFM	Impervious Surfaces	Building	Low Vegetation	Tree	Car	mIoU (%)	mF1 (%)
ours	✓	✓	✓	76.98	84.00	59.71	72.84	67.53	72.21	83.64
w/o SFM	✓	✓		75.86	83.42	59.19	72.62	63.36	70.89	82.65
w/o DCM		✓	✓	75.01	82.90	58.45	72.56	59.71	69.73	81.83
w/o DCM & SFM		✓		74.03	82.47	58.23	72.08	57.54	68.87	81.17
w/o MSCB & SFM	✓			74.18	82.65	58.03	72.11	58.93	69.18	81.41
Baseline (U-Net)				73.25	82.03	57.42	71.89	54.01	67.72	79.90

**Table 4:** Capacity-controlled comparison on the Vaihingen dataset. Bold values indicate the best-performing results.

Method	Params (M)	GFLOPs	mIoU (%)	Average F1 (%)
U-Net	22.70	10.72	67.72	79.90
Capacity-matched U-Net	50.08	23.74	69.14	81.26
SEMU-Net	50.41	72.67	**72.21**	**83.64**

**Table 5:** Comparison of model complexity and segmentation accuracy on the Vaihingen dataset. Bold values indicate the best-performing results, and underlined values represent the second-best performance.

Method	Index		Accuracy (%)	
	Params (M)	GFLOPs	mIoU (%)	Average F1 (%)
UNet	22.7	10.72	67.72	79.90
PSPNet	46.71	18.94	67.83	80.05
DeeplabV3+	38.48	20.78	68.06	80.22
UNetFormer	11.72	5.57	68.80	81.05
CMT	57.22	11.85	69.76	81.78
A2-FPN	12.16	3.16	69.97	81.92
ST-UNet	160.97	78.69	70.06	82.02
SCG-TransNet	41.98	52.97	69.89	81.98
ESMS-Net	328.57	84.55	70.53	82.45
AFENet	20.23	12.50	71.62	83.20
Ours	50.41	72.67	**72.21**	**83.64**

## Data Availability

The original contributions presented in this study are included in the article. Further inquiries can be directed to the corresponding author.
